# Burden of viral hepatitis caused by specific aetiologies in China, 1990–2016: findings from the GBD 2016

**DOI:** 10.1186/s12889-020-09533-4

**Published:** 2020-09-29

**Authors:** Man Li, Zhuo-qun Wang, Lu Zhang, Hao Zheng, Mai-geng Zhou, Dian-wu Liu

**Affiliations:** 1 Department of Epidemiology and Statistic, Hebei Medical University, Shijiazhuang, 050017 Shijiazhuang, People’s Republic of China; 2 Hebei Province Key Laboratory of Environment and Human Health, Shijiazhuang, China; 3 National Center for Chronic and Noncommunicable Disease Control and Prevention, China CDC, 100050 Beijing, People’s Republic of China; 4 Hebei Chest Hospital, 050042 Shijiazhuang, People’s Republic of China

**Keywords:** Hepatitis, Epidemiology, Incidence, Prevalence, Disability-adjusted life year

## Abstract

**Background:**

The aim of this study is to quantify the burden caused by viral hepatitis in China from 1990 to 2016.

**Methods:**

Data from the GBD 2016 study were extracted to calculate incidence, prevalence and disability-adjusted life years (DALYs). Trends in DALYs were assessed in 33 provinces/regions.

**Results:**

From 1990 to 2016, the total incidence of hepatitis decreased by 88.5%. However, the prevalence of hepatitis (counts in thousands), increased by 37.6% from 153,856 (95% UI: 136,047-172,319) in 1990 to 211,721 (95% UI: 179,776-240,981) in 2016, with age-standardized prevalence rates changing slightly. The number and age-standardized rates of prevalence increased by 35.9 and 1.6% for hepatitis B, respectively, and by 81.8 and 30.4% for hepatitis C. Guangxi, Guangdong and Hainan had the highest age-standardized prevalence rates (≥16,500 per 100,000). Tibet, Qinghai and Gansu had the highest age-standardized DALYs rates (≥40 per 100,000). The largest absolute number of DALYs was observed in the 15–49 year age group in 2016. The highest rate of DALYs occurred in males aged 50–69 years and in females aged ≧70 years.

**Conclusion:**

The incidence and DALYs of viral hepatitis decreased dramatically from 1990 to 2016. However, the prevalence still remains at a high level, which may result in heavy burdens in the future.

## Background

Viral hepatitis, which results from inflammation of the liver, is a major public health concern [1–4]. Viral hepatitis can be caused by a series of viruses, namely, hepatitis A virus (HAV), hepatitis B virus (HBV), hepatitis C virus (HCV), hepatitis D virus (HDV), and hepatitis E virus (HEV). HBV and HCV frequently cause chronic hepatitis, which can lead to progressive cirrhosis and to primary liver cancer. World Health Organization (WHO) estimated that 1 in 3 people throughout the world had been infected by HBV or HCV [5] in 2015 and 640 million people had been infected globally in 2016. Among these, 304.6 million were new cases. Approximately 159 million people are infected with acute HAV and 119 million people are infected with HBV [6]. Viral hepatitis causes approximately 1.5 million deaths and significantly affects the quality of life of hundreds of millions of people at the global level [7].

China is a high epidemic area of hepatitis: in the past few decades, China has made great efforts to prevent and control infectious diseases, including hepatitis, and has achieved good results, with the incidence of hepatitis dropping dramatically. However, due to the large population base in China, there are still many cases of hepatitis. China’s legal infectious disease reporting system has found that more than 1.3 million cases of viral hepatitis are diagnosed annually, accounting for one third of the total reported cases. Viral hepatitis ranks first among infectious diseases, of which hepatitis B accounts for 80% of all hepatitis cases. In addition to seriously threatening human health, viral hepatitis also brings heavy economic burdens to patients. The direct economic loss caused by hepatitis B in China is at least 500 billion RMB every year. The medical costs associated with the management of chronic hepatitis B in a few areas in China have been reported [8, 9]. Despite the large burden of disease, investments in hepatitis remain limited at the national and international levels when compared with some other major infectious diseases.

In this study, we first presented a systematic analysis of four kinds of viral hepatitis at the age-sex-provincial level by using updated data from the Global Burden of Diseases, Injuries, and Risk Factors Study (GBD2016), to estimate the incidence and prevalence of hepatitis and calculate years of life lost (YLLs), years lived with disability (YLDs) and disability-adjusted life years (DALYs). Therefore, our study can provide basic information on the current status of hepatitis for policy decision-makers to identify high-risk populations and regions and to make reasonable allocations of the limited funds for prevention and treatment.

## Methods

All data used in this study were obtained from the GBD 2016, which covered 195 countries and territories between 1990 and 2016. The incidence, prevalence, YLLs, YLDs and DALYs for hepatitis from 33 provinces/regions were analyzed, including the Hong Kong and Macao Special Administrative Regions (SAR). The Socio-Demographic Index (SDI) levels of the 33 provinces/regions were classified into high-middle SDI, middle SDI and low-middle SDI according to the SDI value estimated from GBD 2016.

The definitions of acute hepatitis A, hepatitis B, hepatitis C, and acute hepatitis E were infection with the corresponding virus resulting in anti-HAV IgG, HBsAg, anti-HCV IgG, and anti-HEV IgG seroconversion, respectively, regardless of symptoms. All ICD-10 codes under the headings B15, B16, B17.0, B19.1, B35.3, B17.1, B19.2 and B17.2 were included.

Details of the methodology used in the GBD studies in general and the main changes incorporated into the GBD 2016 methods, have been described previously [6, 10]. In brief, DisMod-MR 2.1, a Bayesian meta-regression tool, was used as the main method of estimation in order to ensure consistency between incidence, prevalence, remission, and cause of death rates for each condition. DALYs were calculated as the sum of YLLs and YLDs for each cause, location, age group, sex, and year. YLDs were the number of years living with a disability multiplied by a disability weighting, which was derived from general population-based surveys [11] and reflected the severity of the disability. YLLs were calculated by multiplying the number of deaths from each cause in each age-group by the reference life expectancy at the average age of death for those who die in that age group. Age-standardized rates were computed using the world standard population developed for the GBD study [12]. Spearman correlation was used to relate the SDI index with DALYs.

The 95% uncertainty interval (UI) for each quantity used in the analyses was estimated by taking 1000 samples from the posterior distribution of each quantity, and using the 25th- and 975th-ordered draws of the uncertainty distribution.

## Results

### Incidence and prevalence

We used over 110,000 outcomes of incidence, prevalence, YLD, YLL and DALYs for hepatitis and the following three levels: 29 age groups; 33 provinces/regions; and 7 individual years from 1990 to 2016.

From 1990 to 2016, the incidence of hepatitis in China (counts in thousands) decreased by 88.5%, from 117,492 (95% UI: 66,517-151,302) to 13,502 (95% UI: 10,918-15,967), and age-standardized incidence rates per 100,000 decreased by 87.1% from 10,114 (95% UI: 5734-12,988) to 1305 (95% UI: 950–1549). However, the prevalence of hepatitis in China (counts in thousands) increased by 37.6%, from 153,856 (95% UI: 136,047-172,319) in 1990 to 211,721 (95% UI: 179,776-240,981) in 2016, and age-standardized prevalence rates per 100,000 changed slightly by − 3.3%, from 13,537 (95% UI: 12,075-15,156) to 13,089 (95% UI: 11,882-15,760) (Table 1).

**Table 1:** Incidence, prevalence and DALYs for hepatitis by aetiology

		Incidence		Prevalence		DALYs	
		Count in thousands	Age-standard rate per 100,000	Count in thousands	Age-standard rate per 100,000	Count in thousands	Age-standard rate per 100,000
Hepatitis	1990	117,492 (66517–151,302)	10,114 (5734–12,988)	153,856 (136047–172,319)	13,537 (12075–15,156)	1403.8 (1319.6–1507.7)	135.2 (127.2–145.0)
	2016	13,502 (10918–15,967)	1305 (950–1549)	211,721 (179776–240,981)	13,089 (11,882,15,760)	302.0 (287.1–319.7)	19.6 (18.6–20.8)
	Change (%)	−88.5	−87.1	37.6	−3.3	− 78.5	−85.5
Acute hepatitis A	1990	84,988 (32621–117,655)	7216 (2767–9983)	6538 (2509–9050)	555 (213–768)	220.4 (188.0–257.8)	18.9 (16.1–22.0)
	2016	4522 (2350–5778)	648 (298–848)	348 (181–444)	50 (23–65)	14.1 (12.1–16.5)	1.8 (1.5–2.1)
	Change (%)	− 94.7	−91.0	− 94.7	− 91.0	−93.6	−90.5
Hepatitis B	1990	28,886 (23305–35,481)	2577 (2121,3117)	122,212 (105637–140,054)	10,605 (9216–12,150)	970.8 (898.0–1059.6)	97.8 (90.8–106.3)
	2016	8025 (6304–10,036)	547 (441–672)	166,042 (134231–194,796)	10,777 (8786–12,645)	263.6 (248.2–279.9)	15.7 (14.9–16.7)
	Change (%)	−72.2	−78.8	35.9	1.6	−72.8	−83.9
Hepatitis C	1990	1180 (1062–1304)	110 (100–122)	24,919 (21906–28,054)	2361 (2088,2640)	14.6 (11.6–18.5)	1.6 (1.2–2.00)
	2016	655 (581–739)	86 (75–99)	45,308 (40742–49,995)	3079 (2760,3406)	4.1 (3.1–5.5)	0.3 (0.2–0.4)
	Change (%)	−44.5	−21.8	81.8	30.4	−71.9	−81.3
Acute hepatitis E	1990	2438 (2208–2701)	209 (192–228)	188 (170–208)	16 (15–18)	198.0 (173.4–225.9)	16.8 (14.8–19.1)
	2016	300 (266–333)	24 (22–27)	23 (20–26)	2.0 (1.7–2.1)	20.2 (17.1–24.0)	1.8 (1.6–2.1)
	Change (%)	−87.7	−88.5	−87.8	−87.5	−89.8	−89.3

Among the four kinds of hepatitis, the incidence and prevalence of acute hepatitis A and acute hepatitis E, along with the age-standardized rates, all decreased dramatically. The case numbers and age-standardized rates of prevalence increased by 35.9 and 1.6% for hepatitis B, respectively, and by 81.8 and 30.4% for hepatitis C.

In 2016, Guangdong province ranked first among the 33 provinces/regions of China in the number of newly infected hepatitis patients with 1,170,000 counts. Tibet, Qinghai and Gansu had the highest age-standardized incidence rates at > 2000/100,000 people; Tibet had the highest of all at 12,563/100,000. Guangxi, Guangdong and Hainan had the highest age-standardized prevalence rates at > 16,500/100,000, whereas Beijing, Hong Kong and Macao had the lowest age-standardized incidence rate and prevalence rate (Table 2).

**Table 2:** Incidence, prevalence and DALYs for hepatitis by province

Region	Incidence				Prevalence				DALYs			
	Count in thousands		Age-standard rate per 100,000		Count in thousands		Age-standard rate per 100,000		Count		Age-standard rate per 100,000	
	1990	2016	1990	2016	1990	2016	1990	2016	1990	2016	1990	2016
China	117,491 (66517–151,000)	13,502 (10918–15,967)	10,114 (5735–12,988)	1306 (951–1549)	153,856 (136047–172,319)	211,720 (179776–240,981)	13,537 (12075–15,156)	13,908 (11881–15,760)	1,403,788 (1319601–1,507,691)	302,026 (287096–319,734)	135.2 (127.2–145.0)	19.6 (18.6–20.8)
Hong Kong SAR	35 (29,43)	22 (20,26)	645 (543–769)	395 (356–447)	673 (578,800)	971 (867,1096)	11,463 (9845–13,539)	11,734 (10472–13,240)	573 (496–665)	259 (211–321)	9.7 (8.4–11.3)	2.8 (2.3–3.4)
Macao SAR	10 (7,12)	3.4 (3.0,3.9)	2698 (1998–3224)	677 (606–753)	42 (36,50)	90 (81,100)	11,852 (10209–13,921)	13,824 (12508–15,340)	178 (147–213)	76 (59–96)	53.9 (44.8–64.8)	10.3 (8.2–12.9)
Anhui	5808 (3130,7345)	492 (400,587)	9161 (5123–11,497)	1087 (8093–1283)	7370 (6496,8353)	8368 (6944,9779)	13,146 (11634–14,781)	13,164 (10980–15,355)	57,418 (48750–67,924)	10,933 (9243–12,916)	111.9 (96.1–130.9)	16.4 (14.0–19.1)
Beijing	163 (124,198)	102 (84,125)	1822 (1257,2281)	478 (363–579)	898 (801,1022)	2277 (1999,2654)	8281 (7417–9380)	8053 (7109–9222)	4234 (3456–5094)	1812 (1477–2180)	39.4 (32.8–46.9)	6.5 (5.4–7.7)
Chongqing	1834 (727,2646)	211 (163,256)	11,469 (4603–16,546)	1218 (715–1581)	1978 (1736,2247)	3886 (3351,4445)	12,545 (11054–14,196)	12,985 (11258–14,926)	19,172 (15693–23,467)	4846 (4059–5711)	125.9 (104.0–153.5)	15.8 (13.3–18.5)
Fujian	1131 (597,1532)	203 (162,250)	3384 (1919–4495)	638 (475–774)	3817 (3332,4310)	5619 (4605,6464)	12,710 (11198–14,300)	13,350 (11064–15,261)	15,450 (13137–18,279)	3121 (2629–3720)	59.2 (50.4–69.6)	7.5 (6.4–9.0)
Gansu	6983 (3540,8998)	458 (345,546)	25,665 (13662–32,789)	2746 (1718–3317)	3195 (2790,3601)	3608 (3142,4183)	13,955 (12308–15,713)	13,248 (11649–15,341)	59,742 (49970–71,301)	11,366 (9667–13,274)	283.9 (240.5–334.3)	41.8 (35.9–48.2)
Guangdong	3507 (2541,4378)	1170 (944,1411)	5859 (4359–7197)	1131 (879–1383)	8919 (7867,9922)	21,323 (17,531,24,275)	15,385 (13621–17,079)	16,728 (13972–18,826)	65,622 (55907–77,563)	22,223 (18940–26,336)	130.9 (110.7–154.3)	18.8 (16.1–22.1)
Guangxi	4132 (2538,5207)	618 (476,739)	9544 (5928–11,938)	1586 (1131–1890)	7717 (679,8543)	9387 (7905,10,563)	18,301 (16235–20,200)	18,670 (15775–21,021)	57,716 (48290–68,872)	12,878 (10820–15,363)	161.8 (135.8–194.1)	25.5 (21.5–30.1)
Guizhou	5251 (2098,7709)	447 (325,551)	13,406 (5998–19,270)	1835 (1057–2400)	4136 (3611,4706)	4616 (3983,5357)	12,795 (11248–14,453)	12,844 (11101–14,930)	55,697 (46740–67,208)	11,774 (9891–14,102)	186.9 (158.8–223.1)	33.0 (27.8–38.9)
Hainan	651 (388,804)	155 (113,186)	9876 (6031–12,150)	2108 (1403–2532)	1038 (912,1157)	1685 (1394,1923)	15,806 (13970–17,538)	16,677 (13907–18,953)	8567 (7274–10,065)	2895 (2419–3488)	154.4 (130.8–179.9)	29.8 (25.2–35.2)
Hebei	2357 (1249,3355)	608 (436,765)	4213 (2201–6048)	995 (625–1298)	5678 (4952,6594)	8381 (7304,9662)	9720 (8518–11,273)	10,404 (9150–11,976)	39,809 (34012–46,550)	12,750 (10671–15,165)	73.5 (62.7–85.7)	15.2 (13.0–17.8)
Heilongjiang	1764 (941,2384)	274 (218,336)	4923 (2646–6668)	966 (681–1193)	4881 (4293,5497)	7122 (6021,8060)	13,768 (12195–15,479)	15,502 (13249–17,546)	25,522 (21600–29,864)	6164 (5127–7266)	80.7 (68.8–93.6)	12.7 (10.8–14.9)
Henan	7423 (4195,9428)	797 (641,944)	8882 (5027–11,246)	1150 (866–1353)	13,243 (11,654,14,811)	15,817 (13,125,17,922)	15,743 (13934–17,536)	16,398 (13641–18,565)	86,809 (73426–100,975)	16,320 (14127–18,818)	115.5 (97.9–134.4)	16.7 (14.5–19.1)
Hubei	5216 (2777,6683)	500 (400,599)	9541 (5117–12,185)	1186 (861–1400)	8493 (7472,94,573)	10,168 (8423,11,539)	15,766 (13924–17,482)	16,476 (13672–18,644)	56,397 (47801–67,880)	10,743 (9105–12,495)	116.6 (99.3–139.3)	16.4 (14.0–18.9)
Hunan	7947 (4592,10,077)	851 (683,1013)	12,299 (7170–15,498)	1598 (1185–1893)	9788 (8625,10,868)	11,788 (9755,13,382)	16,072 (14219–17,806)	16,482 (13705–18,623)	101,822 (86086–119,919)	22,413 (19239–26,334)	185.0 (155.9–218.1)	29.4 (25.4–34.2)
Inner Mongolia	1300 (574,1825)	154 (122,188)	5978 (2653–8396)	857 (565–1070)	2088 (1821,2419)	3235 (2857,3701)	9948 (8744–11,397)	11,084 (9910–12,571)	14,266 (12062–16,873)	3023 (2534–3632)	74.5 (63.8–87.6)	10.4 (8.8–12.2)
Jiangsu	2688 (1477,3618)	421 (346,513)	4365 (2327–5944)	647 (490–788)	8377 (7361,9485)	11,231 (9597,12,901)	12,591 (11132–14,259)	12,416 (10709–14,210)	43,122 (36547–50,781)	8491 (7236–9894)	68.9 (58.5–81.1)	8.8 (7.6–10.1)
Jiangxi	9004 (3390,13,050)	707 (494,881)	18,982 (8033–27,070)	2096 (1211–2725)	5420 (4677,6133)	6492 (5424,7490)	13,996 (12272–15,718)	13,321 (11137–15,323)	88,207 (73767–104,129)	17,845 (15172–21,297)	250.4 (210.4–291.4)	36.9 (31.6–43.8)
Jilin	1880 (1295,2286)	234 (193,284)	7604 (5212–9192)	1152 (888–1344)	3502 (3080,3942)	4891 (4143,5557)	14,122 (12523–15,833)	15,334 (13093–17,401)	32,324 (27580–38,029)	6215 (5291–7363)	145.8 (125.6–171.1)	18.0 (15.6–20.9)
Liaoning	1365 (984,1635)	303 (251,360)	3958 (2719–4784)	962 (749–1120)	5294 (4658,5916)	8187 (6939,9235)	13,503 (11918–15,075)	15,503 (13214–17,477)	26,218 (22379–30,759)	6658 (5587–8017)	72.3 (61.8–84.4)	11.8 (10.2–13.9)
Ningxia	692 (349,891)	70 (54,83)	11,075 (5902–14,086)	1379 (959–1637)	594 (525,671)	986 (857,1139)	12,553 (11148–14,075)	13,560 (11834–15,548)	5070 (4228–6024)	1342 (1137–1574)	112.2 (95.9–130.2)	19.6 (16.7–22.7)
Qinghai	1720 (879,2194)	143 (103,171)	26,998 (15016–33,886)	3671 (2258–4440)	666 (575,757)	883 (769,1020)	13,941 (12273–15,723)	13,679 (12065–15,710)	14,322 (11892–17,351)	3251 (2728–3830)	321.9 (275.8–379.9)	54.0 (45.9–63.0)
Shaanxi	4749 (2172,6730)	511 (377,632)	14,585 (6707–20,649)	1921 (1114–2484)	4120 (3620,4693)	5778 (5061,6608)	12,871 (11391–14,582)	13,737 (12125–15,658)	58,470 (50473–68,441)	13,308 (11184–15,669)	199.8 (172.7–232.0)	31.3 (26.8–36.2)
Shandong	3730 (1901,5135)	641 (503,784)	4984 (2455–6928)	823 (549–1033)	10,539 (9326,11,801)	13,749 (11,251,15,845)	12,805 (11380–14,312)	12,562 (10329–14,404)	57,756 (49465–67,354)	13,459 (11474–15,838)	77.5 (66.5–90.3)	11.7 (10.1–13.7)
Shanghai	326 (235,406)	184 (144,228)	3414 (200–4564)	793 (542–989)	1631 (1435,1823)	4379 (3652,5010)	12,209 (10732–13,610)	13,167 (11108–14,917)	8763 (7423–10,470)	3468 (2901–4154)	64.4 (55.3–76)	10.5 (8.9–12.5)
Shanxi	2932 (1653,3679)	388 (300,461)	9649 (5544–12,037)	1450 (961–1726)	2869 (2499,3325)	4280 (3738,4949)	10,171 (8895–11,794)	10,452 (9191–12,034)	35,410 (30379–41,338)	8490 (7235–9932)	136.2 (116.9–158.8)	20.8 (17.8–24.3)
Sichuan	15,661 (8333,19,791)	773 (628,928)	15,222 (8063–19,269)	1549 (1063–1848)	13,204 (11,651,14,955)	10,714 (9173,12,356)	12,777 (11337–14,434)	12,569 (10835–14,509)	165,224 (140875–193,686)	21,649 (18449–25,665)	170.6 (146.0–199.6)	24.4 (20.9–28.8)
Tianjin	117 (95,138)	71 (59,85)	1640 (1232–1930)	552 (453–642)	805 (706,941)	1838 (1615,2123)	9294 (8187–10,800)	10,519 (9319–12,122)	2541 (2148–2989)	1093 (895–1320)	30.5 (26.0–35.7)	6.2 (5.1–7.3)
Tibet	1094 (583,1387)	267 (161,326)	44,117 (24472–55,357)	12,653 (6607–15,776)	337 (289,384)	450 (387,530)	15,299 (13302–17,338)	13,708 (11984–15,818)	11,920 (10253–13,829)	4387 (3724–5175)	610.73 (52.7–700.6)	151.6 (129.0–179.7)
Xinjiang	1930 (990,2436)	309 (224,368)	9624 (5395–11,988)	1854 (1198–2238)	1924 (1702,2189)	3510 (3063,4002)	12,502 (11092–14,098)	13,830 (12183–15,742)	17,693 (14858–21,296)	4963 (4189–5825)	123.4 (104.4–145.8)	21.1 (18.1–24.6)
Yunnan	7507 (4008,9589)	664 (519,791)	15,910 (9073–20,066)	1893 (1318–2251)	4921 (4324,5579)	6740 (5818,7883)	13,005 (11524–14,727)	13,079 (11356–15,163)	72,530 (61762–86,881)	16,407 (13812–19,625)	207.5 (178.6–245.6)	33.2 (28.2–39.4)
Zhejiang	6583 (3888,8167)	750 (588,895)	17,055 (9826–21,214)	1901 (1294–2278)	5699 (5016,6390)	9271 (7636,10,596)	13,710 (12142–15,353)	13,718 (11431–15,579)	95,211 (81062–112,376)	17,400 (14748–20,447)	247.7 (212.1–293.0)	26.3 (22.7–30.9)

**Fig. 1:**
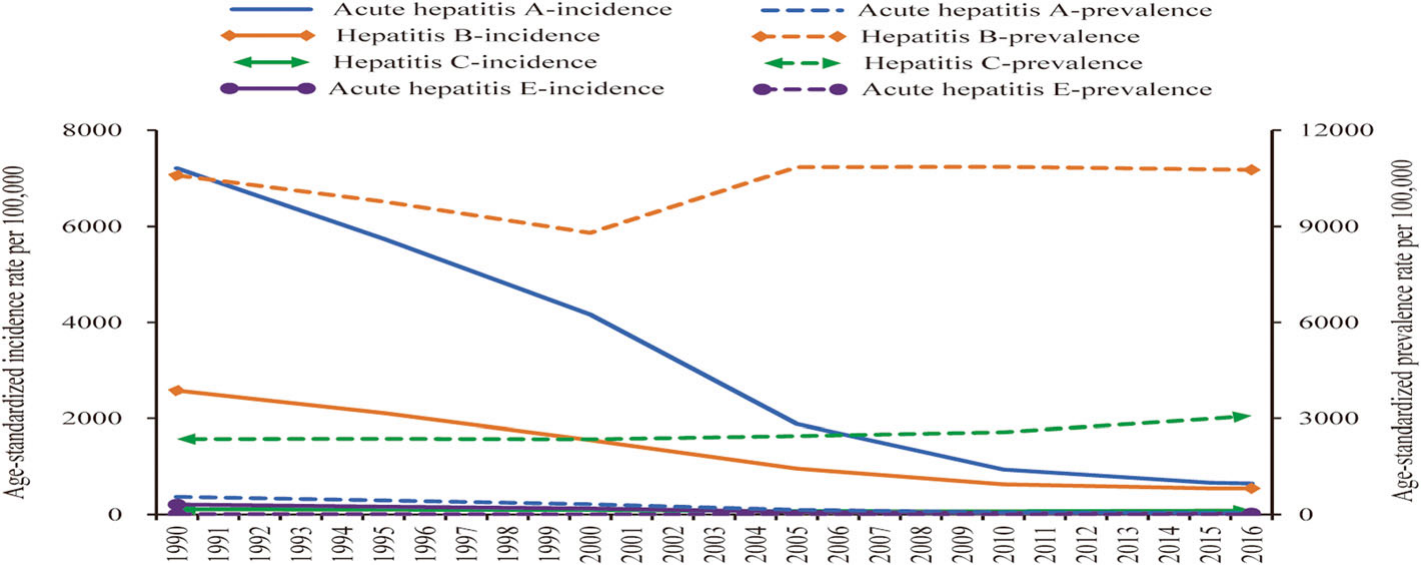
Trends in China from 1990 to 2016 for: (A) all age and age-standardized incidence rates of hepatitis; and (B) all age and age-standardized prevalence rate of hepatitis

Among the four kinds of viral hepatitis, the incidence rates for acute hepatitis A, hepatitis B, and acute hepatitis E decreased dramatically over the last few decades, especially acute hepatitis A and hepatitis B (Fig. 1). However, the age-standardized prevalence rate for hepatitis B decreased slightly from 1990 to 2000 then increased from 2005 and sustained a high level in the past 10 years.

**Fig. 2:**
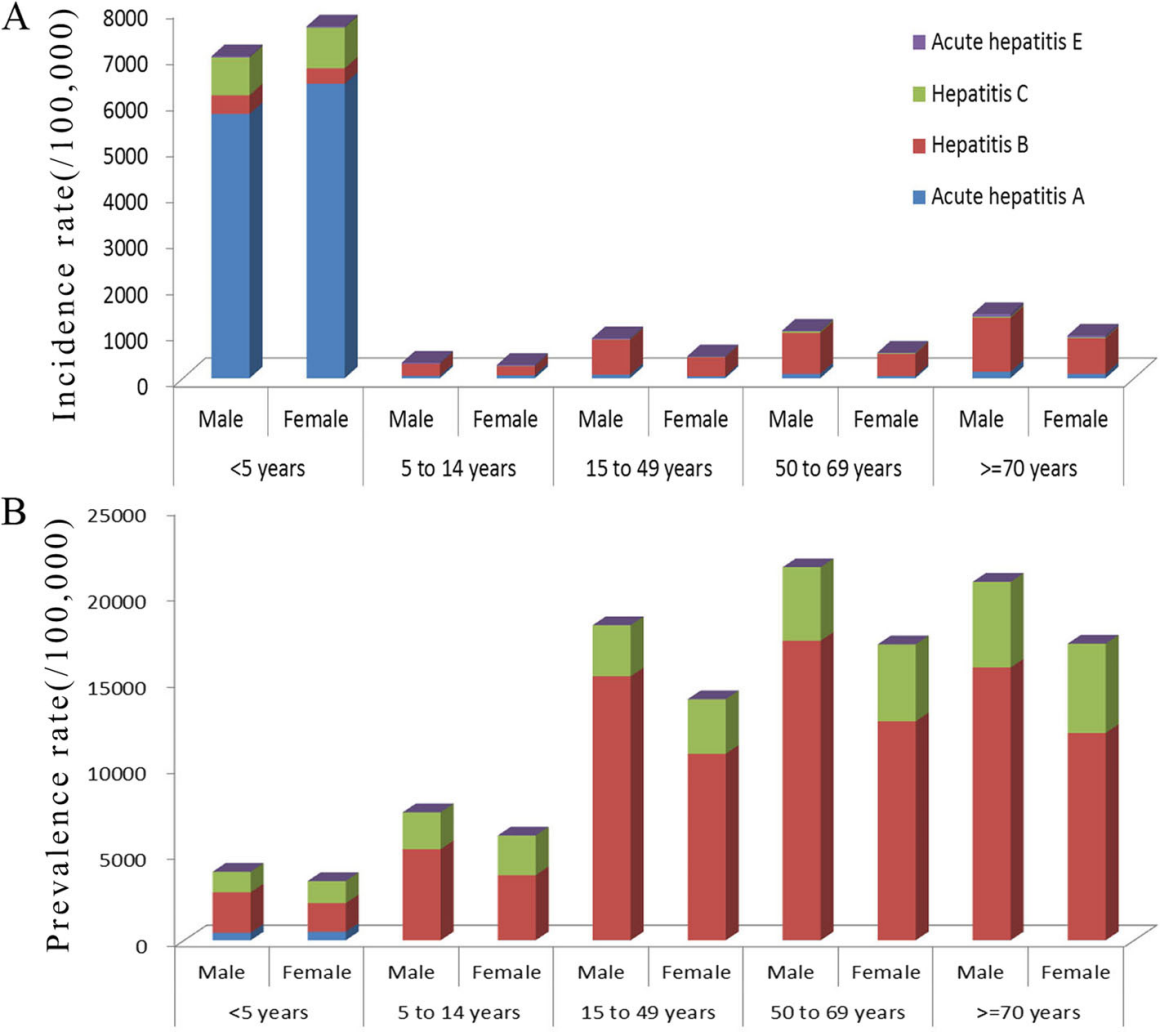
Trends in China in 2016 for: (A) incidence rates of hepatitis by sex and age-group; and (B) prevalence rate of hepatitis by sex and age group

The incidence rates and prevalence rates of the four kinds of hepatitis by sex and age group in 2016 are presented in Fig. 2. The low age group (< 5 year) had the highest incidence rate. For example, the incidence rate of acute hepatitis A was 5733/100,000 for males and 6381/100,000 for females and the incidence rate of acute hepatitis C was 815/100,000 for males and 884/100,000 for females. In most of the age groups, the incidence rates in males were higher than those in females. The prevalence rates slightly increased starting from the < 5 year age group and then sharply increased at 15–49 year age group. The prevalence rates for males and females stabilized starting from the 50–69 year age group.

### DALYs

The DALYs for hepatitis decreased by 78.5%, from 1403,788 (95% UI: 1,319,601-1507,691) in 1990 to 302,026 (95% UI: 287,096-319,734) in 2016. In 2016, the DALYs for acute hepatitis A, hepatitis B, hepatitis C and acute hepatitis E were 14.1, 263.6, 4.1 and 20.2 (in thousands), respectively. The overall age-standardized DALY rate decreased by 85.5% from 135.2 (95% UI: 127.2–1145.0) to 19.6 (95% UI: 18.6–20.8) per 100,000 (Table 1).

Provincial-level DALYs and age-standardized rates of DALYs per 100,000 in 1990 and 2016 are also shown in Table 2. In 2016, the top three provinces with the heaviest disease burden caused by viral hepatitis were Hunan, Guangdong and Sichuan, with the number of DALYs > 21,000. Tibet, Qinghai and Gansu had the highest age-standardized DALY rates at > 40/100,000 people. However, Hong Kong, Beijing, and Tianjin had the lowest age-standardized DALY rates.

**Fig. 3:**
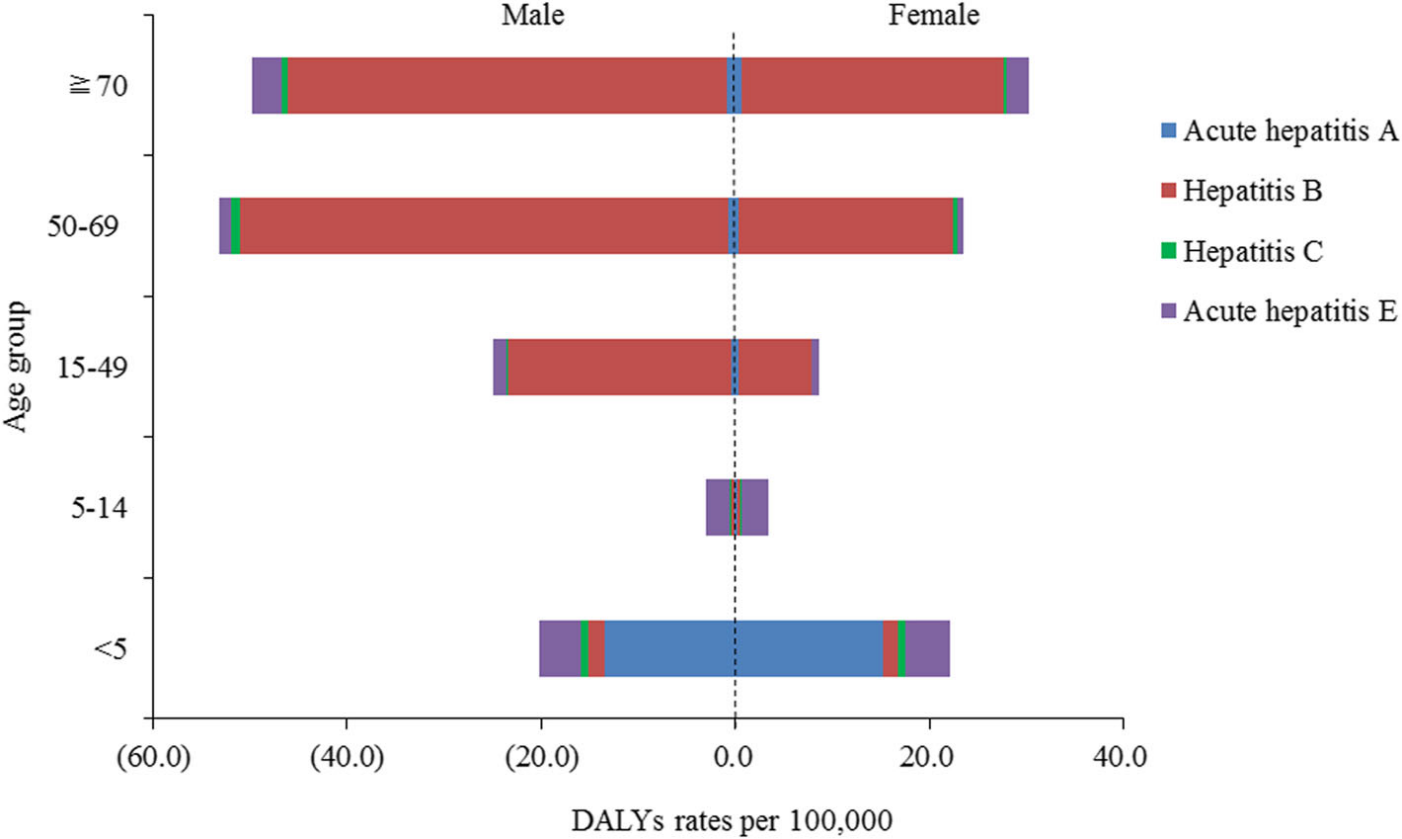
Sex difference in DALY rates per 100,000 for four etiologies of viral hepatitis by age group in 2016

DALYs number and DALY rates in 2016 by sex and age group are shown in Table 3 and Fig. 3. In the < 5 year age group, the burden of disease was mainly caused by acute hepatitis A, with the number of DALYs and age-standardized DALY rate of 8673 (95% UI: 7320–10,839) and 14.3 (95% UI: 12.1–17.9)/100,000, respectively. The overall number of DALYs for viral hepatitis gradually increased starting from the 5–14 year age group and peaked at the 50–69 year age group for male and ≧70 year age group for female. The DALY rates in males were higher than those in females except that in < 5 year age group.

**Table 3:** DALYs and age-standardized DALYs rates in 2016 by aetiology and age-group

Characteristic	DALY in count				DALYs rates (per 100,000)			
	Acute hepatitis A	Hepatitis B	Hepatitis C	Acute hepatitis E	Acute hepatitis A	Hepatitis B	Hepatitis C	Acute hepatitis E
Sex								
male	7918 (6667–9434)	188,799 (176058–202,826)	2638 (1912–3704)	12,035 (9874–14,674)	1.8 (1.5–2.2)	22.1 (20.7–23.8)	0.4 (0.3–0.5)	2.0 (1.7–2.3)
female	6191 (5199–7367)	74,800 (69795–80,525)	1440 (1064–1910)	8203 (6914–9826)	1.8 (1.5–2.1)	9.2 (8.6–9.9)	0.2 (0.2–0.3)	1.6 (1.4–1.9)
Age-group								
< 5	8673 (7320–10,839)	980 (510–1665)	472 (228–920)	2654 (2254–3292)	14.3 (12.1–17.9)	1.6 (0.8–2.7)	0.8 (0.4–1.5)	4.4 (3.7–5.4)
5 to 14	321 (223–454)	509 (328–742)	151 (108–203)	4089 (3609–4672)	0.2 (0.1–0.3)	0.3 (0.2–0.5)	0.1 (0.1–0.1)	2.6 (2.3–3.0)
15 to 49	2713 (1989–3592)	115,597 (107488–124,995)	1085 (746–1529)	8039 (6003–10,422)	0.4 (0.3–0.5)	15.5 (14.4–16.8)	0.1 (0.1–0.4)	1.1 (0.8–1.4)
50 to 69	1767 (1246–2446)	116,715 (109037–124,962)	1931 (1171–3045)	3254 (1887–5270)	0.6 (0.4–0.8)	36.4 (34.0–38.9)	0.6 (0.4–1.0)	1.0 (0.6–1.6)
≧70	634 (439–874)	29,798 (27195–32,331)	440 (267–684)	2202 (1366–3398)	0.8 (0.5–1.0)	35.5 (32.4–38.5)	0.5 (0.3–0.8)	2.6 (1.6–4.1)

According to the SDI value in 2016, the 33 provinces were classified into three groups. The mean number of DALYs for hepatitis B in 2016 was 5970 for the high-middle SDI region, 8533 for the middle SDI region, and 7828 for the low-middle SDI region. The age-standardized DALY rates for hepatitis of the four aetiologies all decreased dramatically after 2000. In 2016, the age-standardized DALY rates for hepatitis B were approximately 9.1/100,000 for high-middle SDI level regions, 17.4/100,000 for middle SDI level regions and 56.4/100,000 for low-middle SDI level regions (Table. 4). There was a moderate negative correlation between SDI and DALYs (r_s_ = − 0.808, *P* < 0.001).

**Table 4:** The DALYs number and age-standardized DALYs rates per 100,000 of viral hepatitis by SDI in China

Socio-demographic index	Cause	DALY number					Age-standardized DALY rate per 100,000				
		1990	2000	2010	2016	Change from 1990 to 2016(%)	1990	2000	2010	2016	Change from 1990 to 2016(%)
High-middle level	Acute hepatitis A	2892	1639	455	319	−88.9	8	4.5	1.2	0.9	− 88.8
	Hepatitis B	21,067	20,233	7471	5970	−71.7	62.9	41.5	12.7	9.1	−85.5
	Hepatitis C	319	301	105	96	−69.9	1	0.7	0.2	0.3	−70.0
	Acute hepatitis E	3252	2376	666	468	−85.6	8.8	5.3	1.5	1	−88.6
Middle level	Acute hepatitis A	7608	3072	661	440	−94.2	19.4	11.7	2.9	2	−89.7
	Hepatitis B	32,668	24,904	9493	8533	−73.9	101	66.4	21.4	17.4	−82.8
	Hepatitis C	490	398	135	133	−72.9	1.6	1.1	0.3	0.3	−81.2
	Acute hepatitis E	6555	3552	854	630	−90.4	17.8	10.2	2.7	2	−88.8
Low-middle level	Acute hepatitis A	8752	4447	940	531	−93.9	53.7	40.1	14.5	10.8	−79.9
	Hepatitis B	26,240	22,803	8847	7828	−70.2	253.4	195.2	68.1	56.4	−77.7
	Hepatitis C	401	363	121	111	−72.3	4	3.1	1	0.8	−80.0
	Acute hepatitis E	7061	4362	1027	705	−90.0	49.4	32.7	10.1	7.4	−85.0

## Discussion

According to a report from WHO, the Western Pacific (6.2% or 115 million individuals) and African regions (6.1% or 60 million individuals) have the highest prevalence levels of viral hepatitis [13]. With the spread vaccination of HAV/HBV, the incidence of viral hepatitis in China has gone a gentle decline to a steady level but was still at a high level [14] . At the same time, our results showed that the prevalence in China is 211,721 (in thousands) in 2016, which accounts for one third of the world population of hepatitis sufferers [6].

The results of national serum epidemiology survey of viral hepatitis in China in 1992 showed that the prevalence rate of HBsAg in the 1–59 age groups was 9.75% [15]. In 2006, a national epidemiological survey of hepatitis B found that the rate of HBsAg prevalence in the 1–59 age groups dropped to 7.18% [16]. However, in our study, the age-standardized prevalence rates for hepatitis from 1990 to 2016 decreased slightly by 3.3% from 13,537 to 13,089 per 100,000 population, while the age-standardized prevalence rates for hepatitis B increased slightly by 1.6% from 10,605 to 10,777 per 100,000 population. We accounted for differences in factors such as diagnostic criteria, test levels, reporting system, and different standard populations. However, these differences do not prevent us from comparing the 33 provinces’ data, which can help us to judge the burden of viral hepatitis for each province and make the right medical decisions.

HAV, HBV, HCV and HEV are biologically unrelated and have different modes of transmission and natural histories of infection. Hepatitis B and hepatitis C are mainly transmitted through blood, mother-to-child transmission, and sexual transmission, while hepatitis A and E are mainly transmitted through the digestive tract. The prevalence of hepatitis A is mainly related to poor socio-economic conditions including high housing density, poor sanitation system and low water quality [17–19]. The incidence of hepatitis A has dropped significantly in China since the introduction of the vaccine in the 1990s. In China, hepatitis B is one of the major diseases that threaten our health. Although the age-standardized incidence rate of hepatitis B has decreased significantly after the widespread use of the HBV prophylactic vaccine, the prevalence rate remains high. For hepatitis B patients who have established chronic infections, there is lack of effective treatment strategies due to the complicated course, poor prognosis and difficulty in curing the infections. The global prevalence of HCV has been estimated at 1%, which equates to approximately 71 million people [20]. Chronic hepatitis C virus infection varies between 0.6 and 10% depending on geographical location [21]. In Western Europe, the estimated prevalence of hepatitis C is 1.5–3.5% [4]. In China, HCV infection is a commonly reported disease. Indeed, China has the largest HCV-infected population in terms of absolute number [22]. In our study, the prevalence of hepatitis C in 2016 was approximately 45 million and increased by 81.8% compared with the prevalence in 1990. Other investigations report similarly rising trends [23]. There are two possible reasons for this trend. First, it may be due to lack of effective vaccines. There are safe and effective vaccines to prevent HAV and HBV, and one vaccine for HEV is commercialized for use only in China, but there are no official approved vaccines for HCV. Second, it may be due to increased and available HCV diagnostics over time [24, 25].

Although the age-standardized prevalence rate in China remains at a high level, the DALY rate had sharply declined by 85.5% from 1990 to 2016. This could be partly explained by the improved antiviral therapy, especially for HBV and HCV. The clinical course of HBV and HCV can be acute and chronic. The clinical manifestations of hepatitis can be absent or appear when the disease is advanced [26]. There was no disability weightings used for asymptomatic acute hepatitis A, B, C or E cases or chronic hepatitis B or C cases [10]. Similar trends for prevalence and DALYs could be observed in most provinces. The top five provinces suffering a heavy burden of hepatitis were Tibet, Qinghai, Gansu, Jiangxi and Yunnan. To realize the “2030 Agenda for Sustainable Development Goals” of WHO [27] and support national hepatitis elimination plans, we should provide adequate funding for those provinces.

HAV and HEV cause only acute hepatitis and are transmitted mostly through exposure to contaminated food or water. In our analysis, although the disease burden caused by hepatitis A decreased dramatically from 1990 to 2016, the percentage of disease burden caused by hepatitis A was still high in the low age group population. It is necessary to recommend vaccination to all children aged 1 year and older [19]. In China, thanks to the universal HBV immunization programme for newborn babies initiated in 1992, the prevalence of anti-HBs was higher in fully immunized children (63.2–74.3%) than in non-immunized children (21.1–34.8%) [28]. The burden of hepatitis B in 2016 decreased dramatically with the total DALYs peaking in the 5069 year age group. The age-standardized prevalence rate in males was higher than that in females, which was also represented in other reports [29–31].

Our study provided the most up-to-date systematic analysis on the disease burden of hepatitis in China. We can not only compare the prevalence, incidence, and DALY rate to other countries, but also compare them at the provincial level. The data used in the study were mainly from the GBD2016 study. To improve model validity and decrease uncertainty from various sources, GBD allows annual improvement to the methods and available data sources. However, limitations still remained [6, 10, 32], which were also applied in our study. First, severity distribution data across sequelae for most diseases were from high-income countries, which might lead to underestimation of YLDs. Second, data sources from national surveys, cancer registries, and surveillance systems of the Disease Control and Prevention could not represent the overall trend of prevalence and DALYs. Third, viral hepatitis were defined on the basis of HAV IgG, HBsAg, HCV Ab, and HEV IgG which do not clearly distinguish acute viral hepatitis, chronic viral hepatitis, versus resolved viral hepatitis. At last, the disease burden of HDV was not evaluated in the GBD2016 and the current study.

## Conclusion

The burden of hepatitis decreased rapidly in China from 1990 to 2016. However, the prevalence rates of hepatitis B and C remained high, with large gaps among provinces and populations. The challenges of communicable disease control persist. Especially for hepatitis B, effective treatment should be carried out to prevent chronic patients from developing cirrhosis and liver cancer, which may exert a large burden on society in the future.

## Data Availability

Data at national level can be found at: http://ghdx.healthdata.org/gbd-results-tool. All subnational level data used and analysed during the current study are available from the corresponding author on reasonable request.

## Abbreviations

DALYs: Disability-adjusted life years
GBD2016: The global burden of diseases, injuries, and risk factors study 2016
HAV: Hepatitis A virus
HBV: Hepatitis B virus
HCV: Hepatitis C virus
HDV: Hepatitis D virus
HEV: Hepatitis E virus
SDI: Socio-demographic Index
UI: Uncertainty interval
YLLs: Years of life lost
YLDs: Years lived with disability

## References

[1] Jefferies M, Rauff B, Rashid H, Lam T, Rafiq S (2018). Update on global epidemiology of viral hepatitis and preventive strategies. World J Clin Cases.

[2] Popping S, El-Sayed M, Feld J, Hatzakis A, Hellard M, Lesi O, Ninburg M, Ward J, Boucher C (2018). Report from the international viral hepatitis elimination meeting (IVHEM), 17-18 November 2017, Amsterdam, the Netherlands: gaps and challenges in the WHO 2030 hepatitis C elimination framework. J Virus Erad.

[3] Chen DS, Locarnini S, Wait S, Bae SH, Chen PJ, Fung JY, Kim HS, Lu SN, Sung J, Tanaka J (2013). Report from a viral hepatitis policy forum on implementing the WHO framework for global action on viral hepatitis in North Asia. J Hepatol.

[4] Modin L, Arshad A, Wilkes B, Benselin J, Lloyd C, Irving WL, Kelly DA (2019). Epidemiology and natural history of hepatitis C virus infection among children and young people. J Hepatol.

[5] Hajarizadeh B, Grebely J, Dore GJ (2013). Epidemiology and natural history of HCV infection. Nat Rev Gastroenterol Hepatol.

[6] Disease GBD, Injury I, Prevalence C (2017). Global, regional, and national incidence, prevalence, and years lived with disability for 328 diseases and injuries for 195 countries, 1990-2016: a systematic analysis for the global burden of Disease study 2016. Lancet.

[7] Lanini S, Pisapia R, Capobianchi MR, Ippolito G (2018). Global epidemiology of viral hepatitis and national needs for complete control. Expert Rev Anti-Infect Ther.

[8] Lu J, Xu A, Wang J, Zhang L, Song L, Li R, Zhang S, Zhuang G, Lu M (2013). Direct economic burden of hepatitis B virus related diseases: evidence from Shandong, China. BMC Health Serv Res.

[9] Xiao J, Lin H, Liu T, Zeng W, Li X, Shao X, Tan Q, Xu Y, Xu X, Zheng H (2015). Disease burden from hepatitis B virus infection in Guangdong Province, China. Int J Environ Res Public Health.

[10] Collaborators GBDCoD (2017). Global, regional, and national age-sex specific mortality for 264 causes of death, 1980-2016: a systematic analysis for the global burden of Disease study 2016. Lancet.

[11] Salomon JA, Haagsma JA, Davis A, de Noordhout CM, Polinder S, Havelaar AH, Cassini A, Devleesschauwer B, Kretzschmar M, Speybroeck N (2015). Disability weights for the global burden of Disease 2013 study. Lancet Glob Health.

[12] Mortality GBD (2016). Causes of death C: global, regional, and national life expectancy, all-cause mortality, and cause-specific mortality for 249 causes of death, 1980-2015: a systematic analysis for the global burden of Disease study 2015. Lancet.

[13] Global Hepatitis Report 2017. Geneva: World Health Organization; 2017. Licence: CC BY-NC-SA 3.0 IGO. ISBN 978-92-4-156545-5.

[14] Xiaochang L, Ting Z, Zhimei Z, Jingdong W, Jiawei L, Wen Y, Jingsi Y (2018). Incidence and trends of viral hepatitis in China. Prev Med (in Chineses).

[15] Wang FS, Fan JG, Zhang Z, Gao B, Wang HY (2014). The global burden of liver disease: the major impact of China. Hepatology.

[16] Liang X, Bi S, Yang W, Wang L, Cui G, Cui F, Zhang Y, Liu J, Gong X, Chen Y (2009). Epidemiological serosurvey of hepatitis B in China--declining HBV prevalence due to hepatitis B vaccination. Vaccine.

[17] WHO (2012). WHO position paper on hepatitis a vaccines - June 2012. Wkly Epidemiol Rec.

[18] Aggarwal R, Goel A (2015). Hepatitis a: epidemiology in resource-poor countries. Curr Opin Infect Dis.

[19] Linder KA, Malani PN (2017). Hepatitis a. JAMA.

[20] Polaris Observatory HCVC (2017). Global prevalence and genotype distribution of hepatitis C virus infection in 2015: a modelling study. Lancet Gastroenterol Hepatol.

[21] Pawlowska M, Sobolewska-Pilarczyk M, Domagalski K (2018). Hepatitis C virus infection in children in the era of direct-acting antiviral. World J Gastroenterol.

[22] Lavanchy D. Evolving epidemiology of hepatitis C virus. Clin Microbiol Infect. 2011;17(2):107–15. 10.1111/j.1469-0691.2010.03432.x 21091831

[23] Liu Z, Yang Q, Shi O, Ye W, Chen X, Zhang T (2018). The epidemiology of hepatitis B and hepatitis C infections in China from 2004 to 2014: an observational population-based study. J Viral Hepat.

[24] Man John Law L, Landi A, Magee WC, Lorne Tyrrell D, Houghton M (2013). Progress towards a hepatitis C virus vaccine. Emerg Microbes Infect.

[25] Xue J, Zhu H, Chen Z (2014). Therapeutic vaccines against hepatitis C virus. Infect Genet Evol.

[26] Cruz HM, Barbosa JR, Baima Colares JK, de Moraes Neto AHA, Alencar MFL, Bastos FI, da Mota JC, Carvalho-Costa FA, Ivantes CAP, Lewis-Ximenez LL (2018). Cross-sectional study to determine viral hepatitis knowledge in different urban populations in Brazil. World J Hepatol.

[27] Hutin Y, Low-Beer D, Bergeri I, Hess S, Garcia-Calleja JM, Hayashi C, Mozalevskis A, Rinder Stengaard A, Sabin K, Harmanci H (2017). Viral hepatitis strategic information to achieve elimination by 2030: key elements for HIV program managers. JMIR Public Health Surveill.

[28] Cui F, Li L, Hadler SC, Wang F, Zheng H, Chen Y, Gong X, Hutin YJ, Cairns KL, Liang X (2010). Factors associated with effectiveness of the first dose of hepatitis B vaccine in China: 1992-2005. Vaccine.

[29] Merat S, Rezvan H, Nouraie M, Jafari E, Abolghasemi H, Radmard AR, Zaer-rezaii H, Amini-Kafiabad S, Maghsudlu M, Pourshams A (2010). Seroprevalence of hepatitis C virus: the first population-based study from Iran. Int J Infect Dis.

[30] Boulos D, Goedhuis NJ, Wu J, Baptiste B, Poliquin D, Furseth J, Chan JI, Bolesnikov G, Barichello F, Andonov A (2005). Enhanced surveillance for acute and likely acute hepatitis B in Canada: 1999 to 2002. Can J Infect Dis Med Microbiol.

[31] Shimelis T, Torben W, Medhin G, Tebeje M, Andualm A, Demessie F, Mulu A, Tegbaru B, Gebre-Selassie S (2008). Hepatitis B virus infection among people attending the voluntary counselling and testing Centre and anti-retroviral therapy clinic of St Paul's general specialised hospital, Addis Ababa, Ethiopia. Sex Transm Infect.

[32] DALYs GBD, Collaborators H (2017). Global, regional, and national disability-adjusted life-years (DALYs) for 333 diseases and injuries and healthy life expectancy (HALE) for 195 countries and territories, 1990-2016: a systematic analysis for the global burden of Disease study 2016. Lancet.

